# Performance of oyster mushroom (*Pleurotus ostreatus*) on paddy straw, water hyacinth and their combinations

**DOI:** 10.1016/j.heliyon.2023.e19051

**Published:** 2023-08-16

**Authors:** Sobita Subedi, Nabin Kunwar, Krishna Raj Pandey, Yagya Raj Joshi

**Affiliations:** a Faculty of Agriculture, Agriculture and Forestry University (AFU), Rampur, Chitwan, Nepal; b Department of Agricultural Economics, Institute of Agriculture and Animal Science (IAAS), Nepal

**Keywords:** Biological efficiency, Pileus diameter, Pinhead formation, Stipe length, Substrates, Yield parameters

## Abstract

This study aimed to investigate the performance of oyster mushrooms on different substrate combinations, including rice straw alone, rice straw and water hyacinth (1:1), rice straw and water hyacinth (1:2), and rice straw and water hyacinth (2:1), in Pokhara, Kaski from April to June in 2022. A Completely Randomized Design with four replications was used to analyze the growth and yield parameters of the four different substrate combinations. The data were analyzed using R-Studio. Highly significant results (P > 0.001) were found in all growth and yield parameters. The best response in terms of days to colonization, pinhead formation, and total crop duration was observed with rice straw alone (11.5, 13.25, and 54.00 days, respectively). The stipe length, pileus diameter, and fruiting body per bunch had their highest values (3.44 cm, 5.21 cm, and 34.50 cm, respectively) with rice straw alone, followed by rice straw and water hyacinth in a 2:1 ratio (3.05 cm, 4.63 cm, and 34.25 cm, respectively). The maximum total fresh yield of mushrooms (1.53 kg with 153.16% BE) was observed for rice straw alone, followed by rice straw and water hyacinth in a 2:1 ratio (1.17 kg with 116.5% BE), while the poorest yield (0.76 kg with 76.38% BE) was observed in the case of rice straw and water hyacinth in a 1:2 ratio. The highest profitability was observed for mushroom production using rice straw alone with a B:C ratio of 1.24, followed by using rice straw and water hyacinth in a 2:1 ratio (1.03), while the lowest B:C ratio was observed for rice straw and water hyacinth in a 1:2 ratio (0.74). Overall, the best treatment was found to be rice straw alone, followed by rice straw and water hyacinth in a 2:1 ratio. These results suggest that using rice straw alone or in combination with water hyacinth in a 2:1 ratio can be an effective strategy for oyster mushroom cultivation.

## 1 Introduction

Oyster mushrooms (*Pleurotus* spp.) are currently the second-largest genus of cultivated mushrooms worldwide after *Agaricus bisporus,* owing to a significant increase in their commercial production over the past few decades [1,2]. The oyster mushroom is a type of edible, saprophytic, and lignocellulolytic mushroom that grows in subtropical conditions at temperatures ranging from 16 to 32 °C. It belongs to the class Agaricomycetes, order Agaricales, family Pleurotaceae or Tricholomataceae, genus “*Pleurotus”,* and species “*ostreatus”* [3]. It is a protein and fiber-rich diet that is high in non-starch polysaccharides, low in fat, and has a distinct flavor [4,5]. Most *Pleurotus* species have the capacity to produce lignocellulosic enzymes that can utilize lignin, cellulose, and hemicellulose [6]. Its ease of growing on numerous unfermented lignocellulosic wastes, high yield potential, superior nutritional qualities, and medicinal values all have contributed to its rising popularity [7].

The composition of the substrate, strain, and carbon-to-nitrogen concentration in the growth medium are all crucial factors that affect a fungus' ability to generate enzymes and produce mushrooms [8]. Oyster mushrooms require a higher amount of carbon and less nitrogen, and most substrates need to be supplemented with nitrogen to achieve the optimal carbon:nitrogen (C:N) ratio for mushroom growth [9]. Mushrooms rely on the carbohydrate content of their growing medium for energy and nutrients to grow and flourish [10]. The C:N ratio of the substrate must be species-specific, with higher nitrogen levels speeding up hyphal growth but slowing down the development of the fruiting body [11]. Studies have found that a C:N ratio between 28:1 and 30:1 is ideal for the best yields [12]. Nitrogen-rich substrates have higher mycelium colonization and pinhead formation, while carbon-rich substrates have higher yield and biological efficiency [13]. High C:N ratio substrates require longer composting times, whereas substrates with an initial C:N ratio equal to or less than 55 do not require long composting times [14]. Though commonly grown on rice or wheat straw, it has been reported that they can grow on a variety of lignocellulosic wastes such as sorghum and maize straw, banana pseudostems, cotton stalks, pea shells, plants such as *Parthenium argentatum*, *Sida acuta*, *Lantana camara*, *Cassia sophera*, and *Tephrosia purpurea*, and aquatic weeds such as water hyacinth (*Eichhornia crassipe)*, reed (*Phragmitis*), and cattail [15]. Several studies have shown that various substrates could be used for the cultivation of *Pleurotus* sp. such as coffee pulp, sawdust, cocoa, peanut and coconut shells, cotton seed hulls, jamaica, cassava peels, cotton, sorghum, banana, corn stalks, grass, clover, wood, wastes of rice, rice, sawdust, cotton from textile industry, corn cobs, crushed bagasse and molasses from sugar industry, water hyacinth, water lily, bean, rice straw, leaves, oil-palm fiber, paper and paddy [1,[16], [17], [18], [19], [20], [21], [22], [23]].

Zied et al. [24]; found that using water hyacinth as a substrate for oyster mushroom cultivation in India resulted in a greater total yield and larger mushrooms compared to paddy straw. The total yield was only 10.5 kg in paddy straw, whereas it was 15 kg in water hyacinth. Similarly, Cayetano-Catarino and Bernabé-González [15] also used water hyacinth as a low-cost substrate in combination with rice straw for the cultivation of *Pleurotus* species, which resulted Water hyacinth and rice straw (1:1) supported a significant increase in biological yield in all three species in the first flush at optimal temperatures. No significant differences were obtained on the nutritional composition of mushrooms due to the supplementation of rice straw with water hyacinth, except for the pH and EC. Similar results were obtained by Chang and P. Miles [25] in their investigation of the cultivation of *Pleurotus sajor–cajo* on rice straw and water hyacinth. The best response in the form of pin head appearance and productivity of mushrooms came from the bags containing only rice straw (3.1 kg), followed by the 3:1 combination of rice straw + water hyacinth (2.6 kg), the 1:1 combination of rice straw + water hyacinth (1.9 kg), 1:3 combinations of rice straw + water hyacinth (1.5 kg), and only water hyacinth (0.77 kg). Cueva, Hernández and Niño-Ruiz [26] in his study to obtain a cost-effective production of oyster mushrooms, the invasive aquatic weed water hyacinth had been tried out as a substrate in different combinations with rice straw (1:1, 1:2, and 2:1), also found that a 1:1 combination of rice straw and water hyacinth resulted in a significant increase in mushroom yield, especially in the first flush, and the nutritional qualities of the mushrooms were not significantly affected. These studies suggest that water hyacinth can be safely used as an alternate substrate for oyster mushroom cultivation to reduce production costs and recycle the weed in an eco-friendly way.

Rice is the main agricultural crop in Nepal. The escalating price of the rice straw due to its demand in cattle feeding, thatching, etc., is restricting its availability as a substrate for mushroom cultivation. There is seasonal unavailability and a higher cost of paddy straw in Nepal as compared to water hyacinth (Maharjan, personal communication, October 5, 2021). Therefore, under such circumstances, other cheap yet good substrates for oyster mushroom production must be explored, and that could be water hyacinth. Water hyacinth is one of the world's worst invasive weeds, causing a serious problem in various aquatic bodies that has a negative impact on both humans and animals, the economics of waterways, and agriculture by covering ponds and lakes and invading paddy fields in some areas, rendering them unproductive. A faster rate of weed proliferation is responsible for the premature death of freshwater lakes through eutrophication. On the other hand, it has drawn attention as a plant capable of removing toxic heavy metals (e.g., Cr, Cd, Ni, As, Pb, and Eu) from waste water by adsorbing them on its root and is currently being used in waste water treatment. Among the many control methods for this aquatic weed, the biological method involves using it as a substrate for mushroom cultivation [27]. The problem of this invasive weed might be overcome by blending it with other commonly used wastes like rice or wheat straw, sawdust, etc. for mushroom production. Eradication of this obnoxious weed through the utilization of vast quantities available at no cost is an eco-friendly option, prompting studies on its use for oyster mushroom cultivation. In the context of Nepal, no sufficient research was done previously on the utilization of water hyacinth as a possible alternate substrate. So, there is a strong need for proper investigation on the same issue to further validate these contradictions. The present work has studied the cultivation of *Pleurotus ostreatus* on paddy straw, water hyacinth, and their combinations to assess any significant difference in the growth and yield attributes of mushrooms along with their profitability.

## 2 Materials and methods

### 2.1 Research site

The working site of Horticulture Resource Development Center, Malepatan-5, Pokhara, Kaski was selected as the area of action research. The research site lies under mid-hill subtropical climatic zone with a latitude and longitude of 28 ° 13′03″ N and 83° 58′28″ E respectively and elevation of 854 masl. Pokhara was purposively selected for the experiment as oyster mushroom is intensively produced in Pokhara and also due to the possibility of utilization of aquatic weed i.e. water hyacinth abundantly available in the lakes of Pokhara.

### 2.2 Growing environment

**Fig. 1:**
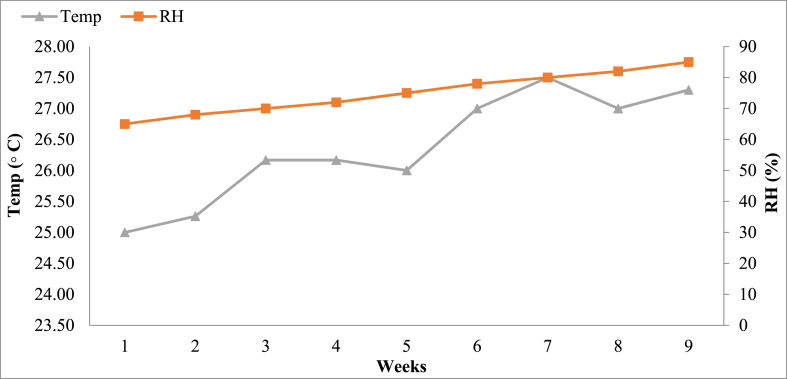
Temperature and relative humidity in the mushroom growing house during the experimental period in Pokhara, Kaski.

The temperature and relative humidity regimes were maintained between 25 and 30 °C, and 65–85%, respectively, during the growing period as shown in Fig. 1 which is ideal temperature for normal growth and development of *P. ostreatus* mushroom. The temperature and relative humidity were in increasing trend during that period.

### 2.3 Sample and sampling technique

A total of 16 mushroom balls were prepared which are all considered as samples in the study. Fruiting bodies were randomly selected for data collection.

### 2.4 Research design

The experimental research was carried out to find out the appropriate combination of substrates for the higher yield of oyster mushrooms. Completely Randomized Design (CRD) was employed to examine the effect of substrate. Four treatments were used in this experiment viz. T1 (Paddy straw alone), T2 (Paddy straw + Water hyacinth in 1:1 ratio), T3 (Paddy straw + Water hyacinth in 1:2 ratio) and T4 (Paddy straw + Water hyacinth in 2:1 ratio). There were four replications for all the treatments. All mushroom balls were randomly placed inside the mushroom house.

Paddy straw and Water Hyacinth were procured from local paddy field and Rupa Lake, Kaski respectively. Paddy straw was chopped into small pieces of size 2″ followed by cleaning and shade drying for 12 h while in case of Water Hyacinth, it was chopped into the same size discarding roots section prior to sun drying for 7–8 days.

### 2.5 Data and data types

Different data were collected from all replications of each treatment i.e. four balls for each treatment and used to examine the effectiveness of the research. Different growth parameters viz. days to colonization, days to pin head formation, stipe length (cm), pileus diameter (cm); yield attributing parameters viz. number of fruiting bodies per bunch, total fresh yield per kg substrate and profitability analysis parameter i.e. BC ratio were recorded under the study.

The study parameters including stipe length, pileus diameter, fruiting body per bunch, and fresh yields were measured at three flushes at 20 Days After Sowing (DAS), 42 DAS and 60 DAS respectively while the total fresh yields were measured during the final harvest.

### 2.6 Data analysis techniques

The data obtained from the experimental plots on various parameters were statistically analyzed to find out the significance of treatments according to the principles of experimental design. Duncan's Multiple Range Test (DMRT) was employed for mean separations to find out the significant differences between the mean values at 5% level of significance (Gomez and Gomez, 1984).

## 3 Results and discussion

Different growth and yield parameters were measured and analyzed for the presence or absence of significant difference between the parameters with different treatments. The results of each parameter have been discussed and interpreted in this section.

### 3.1 Growth parameters

#### 3.1.1 Days to colonization, pinhead formation and total crop duration

##### 3.1.1.1 Days to colonization

T1 had the best response in terms of early mycelial colonization, followed by T4, T2, and T3 with values 11.5, 16, 19.5, and 25 days, respectively as shown in Table 1. There are fewer interspaces in the water hyacinth substrate, which might be the major reason for poor colonization. Also, low cellulose, hemicellulose, and lignin content in water hyacinth may further contribute to poor mycelial development by creating a low C:N ratio [28,29].

**Table 1:** Crop duration of mushroom influenced by various substrates at Pokhara, Kaski, 2022.

Treatment	Days to colonization	Days to pin head formation	Total crop duration (days)
RS	11.5^a^	13.25^a^	54.00^a^
RS + WH (1:1)	19.5^c^	18.75^c^	61.50^c^
RS + WH (1:2)	25.0^d^	20.25^d^	66.25^d^
RS + WH (2:1)	16.0^b^	17.75^b^	56.50^b^
SEM (±)	0.14	0.13	0.16
LSD (0.05)	0.89	0.77	0.96
F-Probability	<0.001	<0.001	<0.001
CV%	3.21	2.86	1.06
Grand Mean	18	17.5	59.56

CV= Coefficient of variation, LSD = Least Significant Difference, SEM= Standard Error of Mean, Mean followed by different letters within columns are significantly different based on DMRT P = 0.05. *** Significant at 0.001 P value.

##### 3.1.1.2 Days to pin head formation

Earliest pinhead formation was observed in T1 (13.25 days), followed by T4 (17.75 days), T2 (18.75 days), and T3 (20.25 days).

##### 3.1.1.3 Total crop duration

The shortest crop duration of mushrooms was reported in T1 (54 days), followed by T4 (56 days), T2 (61.5 days), and T3 (66.25 days), as depicted in Table 1.

Because of more compactness and less interspace for mycelium spreading caused by water hyacinth, the time period for colonization, pinhead formation, and fruiting was longer in treatments with a higher proportion of water hyacinth. This finding was also consistent with Zied et al. [24]; and Chang and Miles [25], who reported delayed appearance of pin heads in water hyacinth alone and in combination with wheat straw. Poor mycelial growth could also be due to poor cellulose, hemicellulose, and lignin content in water hyacinth, causing a low C:N ratio [28,29]. Similarly, these findings are consistent with the findings of [25], who found that the bags containing wheat straw alone produced the best response in terms of pin head appearance, followed by the 3:1 combination of wheat straw + water hyacinth, the 1:1 combination of wheat straw + water hyacinth, the 1:3 combinations of wheat straw + water hyacinth, and water hyacinth only. Poor mycelial growth and its later development could be due to the very high moisture content of water hyacinth and its poor cellulose, hemicellulose, and lignin content, causing a low C:N ratio and rendering a longer crop duration [28,29].

#### 3.1.2 Stipe length pileus diameter and fruiting body per bunch

Similarly, at the 1% level of significance, the study findings on stipe length, pileus diameter, and fruiting body per bunch of oyster mushrooms revealed highly significant results with varied substrates used for mushroom production as shown in Table 2. The best response in the form of stipe length, pileus diameter, and fruiting body were observed for the substrate, rice straw only, with values of 3.44 cm, 5.21 cm, and 34.50 cm, respectively, followed by the 2:1 combination of rice straw + water hyacinth and the 1:1 combination of rice straw + water hyacinth. These parameters were found to be least for 1:2 combinations of rice straw and water hyacinth, as shown in Table 2. Similar study conducted by Elishashvili et al. [30]; divulged similar results regarding highest stipe length and pileus diameter from paddy straw. Cohen, Persky and Hadar [31] concluded that paddy straw contains more carbon to nitrogen (C:N ratio) as compared to water hyacinth but that the CN ratio is more fleeting with alternate harvests as compared to mixtures with water hyacinth due to the enduring CN ratio in mixed substrate.

**Table 2:** Stipe length, pileus diameter and fruiting body per bunch of mushroom influenced by various substrates at Pokhara, Kaski, 2022.

Treatment	Stipe length (cm)	Pileus diameter (cm)	number of fruiting body per bunch
RS	3.44^a^	5.21^a^	34.50^a^
RS + WH (1:1)	2.77^c^	4.90^c^	30.50^b^
RS + WH (1:2)	2.50^d^	4.65^c^	16.50^c^
RS + WH (2:1)	3.05^b^	4.63^b^	34.25^a^
SEM (±)	0.02	0.03	0.59
LSD (0.05)	0.15	0.17	3.66
F-Probability	<0.001	<0.001	<0.001
CV%	3.27	2.27	8.21
Grand Mean	2.94	4.85	28.94

CV= Coefficient of variation, LSD = Least Significant Difference, SEM= Standard Error of Mean, Mean followed by different letters within columns are significantly different based on DMRT P = 0.05. *** Significant at 0.001 P value.

### 3.2 Yield parameters

#### 3.2.1 Yield and biological efficiency

The highest fruiting body yield was obtained from T1 (1531.21 g), followed by T4 (1165.00 g), T2 (952.50 g), and T3 (763.75 g) as shown in Table 3. The yield results were found to be similar to those of [31].

**Table 3:** Yield and biological efficiency of mushroom influenced by various substrates at Pokhara, Kaski, 2022.

Treatment	Yield (g fresh weight/kg dws)			Total Yield (g/kg dws)	Biological Efficiency (%)
	1st Flush	2nd Flush	3rd Flush		
RS	857.5^a^	501.25^a^	172.50^a^	1531.25^a^	153.16^a^
RS + WH (1:1)	590^c^	248.75^c^	113.75^b^	952.50^c^	95.25^c^
RS + WH (1:2)	512^d^	171.25^d^	80.00^c^	763.75^d^	76.38^d^
RS + WH (2:1)	670^b^	327.50^b^	167.50^a^	1165.00^b^	116.50^b^
SEM (±)	6.75	3.77	2.57	9.29	0.93
LSD (0.05)	41.60	23.26	15.84	57.23	5.72
F-Probability	<0.001	<0.001	<0.001	<0.001	<0.001
CV%	4.10	4.84	7.70	3.37	3.37
Grand Mean	657.5	312.18	133.43	1103.13	110.31

CV= Coefficient of variation, LSD = Least Significant Difference, SEM= Standard Error of Mean, Mean followed by different letters within columns are significantly different based on DMRT P = 0.05. *** Significant at 0.001 P value.

**Fig. 2:**
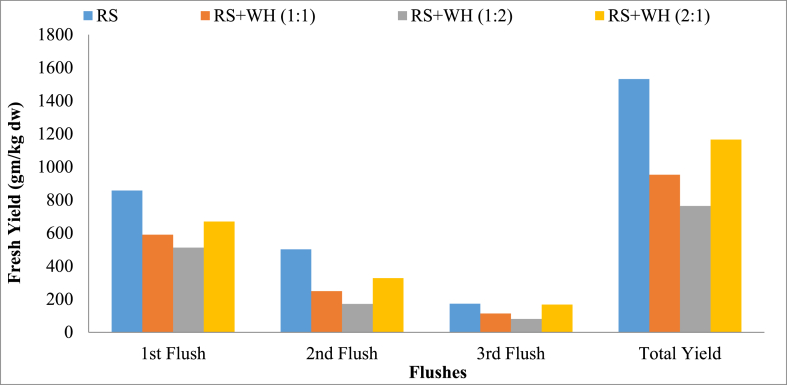
Bar graphs showing the total yield of mushroom (g/kg dws) of *Pleurotus oestratus* after three flushes on each of the four substrate combinations.

From the first to the third flush of harvests, a decreasing trend in yield was observed as shown in Fig. 2. These findings are in line with those of [32,33]. It is due to the decreasing nutrient content of the substrate consumed by mushrooms during growth from one flush stage to the next as a result of mushrooms being lingo-cellulolytic, i.e., breaking and feeding on cell wall contents [34].

It clearly depicts that with an increase in the amount of water hyacinth in the mushroom substrate, there was a decrease in the yield performance of oyster mushrooms, and vice versa. These findings are in accord with those of Mishra and Mishra [18], who reported the highest productivity of mushrooms on wheat straw only (3.1 kg) and the lowest on water hyacinth only (0.77 kg). So, with an almost linearly decreased productivity of the mushroom and an increased ratio of low nutritional value of water hyacinth, i.e., low cellulose content, and a very high moisture content of *E. crassipes* (up to 95%), which discourages better growth and development, resulting in a lower yield. Besides, *Pleurotus* spp. prefer higher cellulose content for their growth and development [12]. For better mushroom production, the substrate must have water-holding capacity [35] and rice straw has a higher water-holding capacity than that of water hyacinth, though it initially has a higher moisture content. Also, the delayed appearance of pin heads affected the final yield of mushrooms in water hyacinth and its combinations with rice straw. The results from Daba et al. [13]; reported that nitrogen-rich substrates, such as bagasse, cereal husks, and sawdust, produce fewer mushrooms than carbon-rich substrates. However, as *Pleurotus* species require high-carbon, low-nitrogen substrates, rice straw, which is rich in carbon source components including lignin, cellulose, and hemicellulose with low nitrogen content, is highly desirable [36]. Besides, poor mushroom growth on water hyacinth-containing substrates could be due to a low C:N ratio. The optimal C:N ratio for the growth of oyster mushrooms is 47.99:1 [37]. Therefore, it is concluded on the basis of the present study that water hyacinth, a troublesome aquatic weed, cannot be exploited as a sole substrate for the production of mushrooms and that its combination with rice straw did not support its higher production.

However, the results of the present study are in contrast with the findings of Gateri et al.; [38], who reported a yield of mushrooms that was almost 1.5 times higher in the case of water hyacinth used as the sole substrate compared to the production on rice straw alone. They emphasized the large-scale use of water hyacinth for mushroom production, on the one hand, and the use of this weed for beneficial purposes, which will also be useful in removing the weed from water bodies, on the other. This could be due to the difference in the species of water hyacinth used or the strain of *P. ostreatus* involved in the studies. The yield is influenced by the nutritional content, physical attributes, and mushroom strain [39]. However, the current study found that increasing the water hyacinth ratio reduced P. *ostreatus* productivity, which could be attributed to a low nutritional value with a high moisture content of E. *crassipes* (up to 95%) discouraging its higher production under study.

Moreover, with the increased ratio of water hyacinth in the substrate, it took more time for the appearance of the pinheads, which subsequently affected the productivity of the mushroom, delaying its harvest period. Therefore, the use of water hyacinth as a sole substrate for mushroom cultivation for better productivity is not recommendable.

### 3.3 Profitability analysis

The economic analysis of mushroom cultivation under a standard tunnel house of size 3033 sq. feet with 330 ball capacity using various substrate combinations revealed that the cost of cultivation of oyster mushrooms was highest in rice straw (NRs. 61,200), followed by rice straw and water hyacinth in a 2:1 ratio (NRs. 56,205), rice straw and water hyacinth in a 1:1 ratio (NRs. 53,705), and rice straw and water hyacinth in a 1:2 ratio (NRs. 51,210) as shown in Table 4.

**Table 4:** Cost of cultivation of mushroom under tunnel of size 30 × 33 sq. feet with 330 balls capacity with various substrates combination in Pokhara, Kaski, 2022.

S.N.	Particulars	Rate (NRs.)	RS		RS + WH (1:1)		RS + WH (1:2)		RS + WH (2:1)	
			Qty.	Total	Qty.	Total	Qty.	Total	Qty.	Total
1	Bamboo	270	25	6750	25	6750	25	6750	25	6750
2	Thread	250	2 Kg	500	2 Kg	500	2 Kg	500	2 Kg	500
3	Silpaulin plastic	6200	1	6200	1	6200	1	6200	1	6200
4	Jute sacks	150	25	3750	25	3750	25	3750	25	3750
5	Plastic sheet	–	–	2500	–	2500	–	2500	–	2500
6	Rice straw	15	1000 Kg	15000	500 Kg	7500	334 Kg	5010	667 Kg	10005
7	Bolls holding thread	–	–	2000	–	2000	–	2000	–	2000
8	Spawn	200	60 balls	12000	60 balls	12000	60 balls	12000	60 balls	12000
9	Labor (spawning + tunnel construction)	6500 + 6000		12500		12500		12500		12500
	Total			61,200		53,700		51,210		56,205

Similarly, the profitability analysis of mushroom production reported a higher B:C ratio in substrate rice straw alone (1.24) and the lowest in substrate rice straw and water hyacinth in a 1:2 ratio (0.74). Rice straw and water hyacinth (2:1) and rice straw and water hyacinth (1:1) showed the B:C ratios of 1.03 and 0.88, respectively, in this study, as shown in Table 5. Profitability analysis was done under the study to determine the economic viability of using a particular substrate for mushroom cultivation. Mushroom cultivation is a business, and profitability is a key concern for growers. Therefore, analyzing the profitability of different substrates can help growers make informed decisions about which substrates to use for cultivation.

**Table 5:** Profitability comparison of mushroom cultivation under various substrate combinations.

Substrates	Cost (NRs.)	Benefit (NRs.)	B:C
RS	61200	75796.88	1.24
RS + WH (1:1)	53700	47148.75	0.88
RS + WH (1:2)	51210	37805.63	0.74
RS + WH (2:1)	56205	57667.5	1.03

## 4 Conclusion

In conclusion, the study found that paddy straw alone produced the highest economic yield performance, followed by a 2:1 ratio of paddy straw and water hyacinth. Paddy straw alone was found to be the best substrate in terms of all parameters, followed by paddy straw and water hyacinth (2:1). The treatment with the lowest ratio of paddy straw and water hyacinth was the least profitable. The study suggests that water hyacinth can be safely mixed with rice straw (1:2) as an alternate substrate for the cultivation of Pleurotus ostreatus, reducing the cost of production while also reprocessing unwanted weed in an environmentally friendly manner. However, water hyacinth alone cannot be used as a sole substrate for mushroom production due to its low nutritional value and high moisture content.

## Funding statement

This research did not receive any speciﬁc grant from funding agencies in the public, commercial, or not-for-proﬁt sectors.

## Author contribution statement

Sobita Subedi: Conceived and designed the experiments; Performed the experiments; Analyzed and interpreted the data; Contributed reagents, materials, analysis tools or data; Wrote the paper.

Nabin Kunwar, Krishna Raj Pandey and Yagya Raj Joshi: Performed the experiments; Analyzed and interpreted the data; Contributed reagents, materials, analysis tools or data; Wrote the paper.

## Data availability statement

Data included in article/supp. material/referenced in article.

## Additional information

Supplementary content related to this article has been publish online at [URL].

## Funding

No funding was received for this work.

## Intellectual property

We confirm that we have given due consideration to the protection of intellectual property associated with this work and that there are no impediments to publication, including the timing of publication, with respect to intellectual property. In so doing we confirm that we have followed the regulations of our institutions concerning intellectual property.

## Research ethics

We further confirm that any aspect of the work covered in this manuscript that has involved human patients has been conducted with the ethical approval of all relevant bodies and that such approvals are acknowledged within the manuscript.

## Authorship

All listed authors meet the ICMJE criteria. We attest that all authors contributed significantly to the creation of this manuscript, each having fulfilled criteria as established by the ICMJE.

We confirm that the manuscript has been read and approved by all named authors.

We confirm that the order of authors listed in the manuscript has been approved by all named authors.

## Contact with the editorial office

The Corresponding Author declared on the title page of the manuscript is:

Sobita Subedi.

This author submitted this manuscript using his/her account in EVISE.

We understand that this Corresponding Author is the sole contact for the Editorial process (including EVISE and direct communications with the office). She is responsible for communicating with the other authors about progress, submissions of revisions and final approval of proofs.

We confirm that the email address shown below is accessible by the Corresponding Author, is the address to which Corresponding Author's EVISE account is linked, and has been configured to accept email from the editorial office of American Journal of Ophthalmology Case Reports: sobitasubedi0306@gmail.com.

We the undersigned agree with all of the above.

## Declaration of competing interest

The authors declare that they have no known competing financial interests or personal relationships that could have appeared to influence the work reported in this paper.
